# Exploring the use of assigned general practitioner and emergency health care in Sami and non-Sami. A repeated cross-sectional study (2012–2019)

**DOI:** 10.1080/02813432.2026.2653008

**Published:** 2026-04-24

**Authors:** Tone Engen, Lena Henriksen, Marita Melhus, Ann-Ragnhild Broderstad, Hans Olav Melberg, Astrid M. A. Eriksen

**Affiliations:** ^a^ Centre for Sami Health Research, UiT The Arctic University of Norway, Tromsø, Norway; ^b^ Department of Nursing and Health Promotion, OsloMet, Oslo, Norway; ^c^ Medical Clinic of Harstad, University Hospital of North Norway, Harstad; ^d^ Department of Community Medicine, UiT The Arctic University of Norway, Tromsø, Norway

**Keywords:** Indigenous, Sami, primary health care, SAMINOR, KUHR-database

## Abstract

**Introduction:**

Indigenous peoples tend to differ from their non-Indigenous counterparts in use of health care services. Lack of available services, socioeconomic disparities, communication barriers and lack of trust have been found to be barriers driving the observed differences.

**Aim:**

The aim of this study is to investigate ethnic differences in the use of assigned general practitioner and emergency health care (2012–2019) while adjusting for age, length of education, and county of residence.

**Methods:**

Data from the SAMINOR 2 Questionnaire Survey was linked to records from the Norwegian Control and Payment of Health Refunds Database. Sami ethnicity was the primary exposure variable. Differences in mean number of consultations were investigated using the two-sample t-test. Adjustments for age (18–69 years), county of residence (Nordland/Trøndelag, Troms, Finnmark) and years of education (≤12 years, >12 years) were done using the generalized linear models Poisson loglinear and Negative binomial with log link.

**Results:**

The study population consisted of 10,694 individuals: 5986 women (28.9% Sami) and 4708 men (22.7% Sami). Sami individuals had a significantly higher mean use of emergency medical care annually, and over the entire study period (2012–2019): Women (Sami 0.51, non-Sami 0.31, *p* < 0.01), Men (Sami 0.40, non-Sami 0.25, *p* < 0.01). However, no significant differences were observed in use of general practitioners. Adjusting for covariates did not alter the results for either outcome.

**Conclusion:**

The findings highlight ethnic disparities in primary healthcare utilization, showing higher use of emergency medical care in Sami compared to non-Sami. Further research should explore the underlying and probably multifaceted factors driving the observed differences.

## Introduction

Indigenous peoples differ in use of health care services compared to their majority population counterparts [1,2]. A systematic review focusing on individuals with arthritis in Canada, Australia and New Zealand revealed significantly higher hospitalisation rates but lower use of specialized care in Indigenous compared to non-Indigenous peoples [3]. Similarly, a recent Australian study exploring frequency, timing and reason for health care utilization found that Aboriginals and Torre Strait Islanders, particularly women, had a higher use of urgent care centres and emergency departments [4]. In Norway, an early investigation done in a multiethnic community in Northern Norway implied a down to 60% lower use of primary health care in Indigenous Sami than in non-Indigenous individuals [5]. Since then, an assumption of a lower use of health care services in the Sami population has been existing alongside research pointing towards slightly poorer health outcomes.

**Figure 1.**
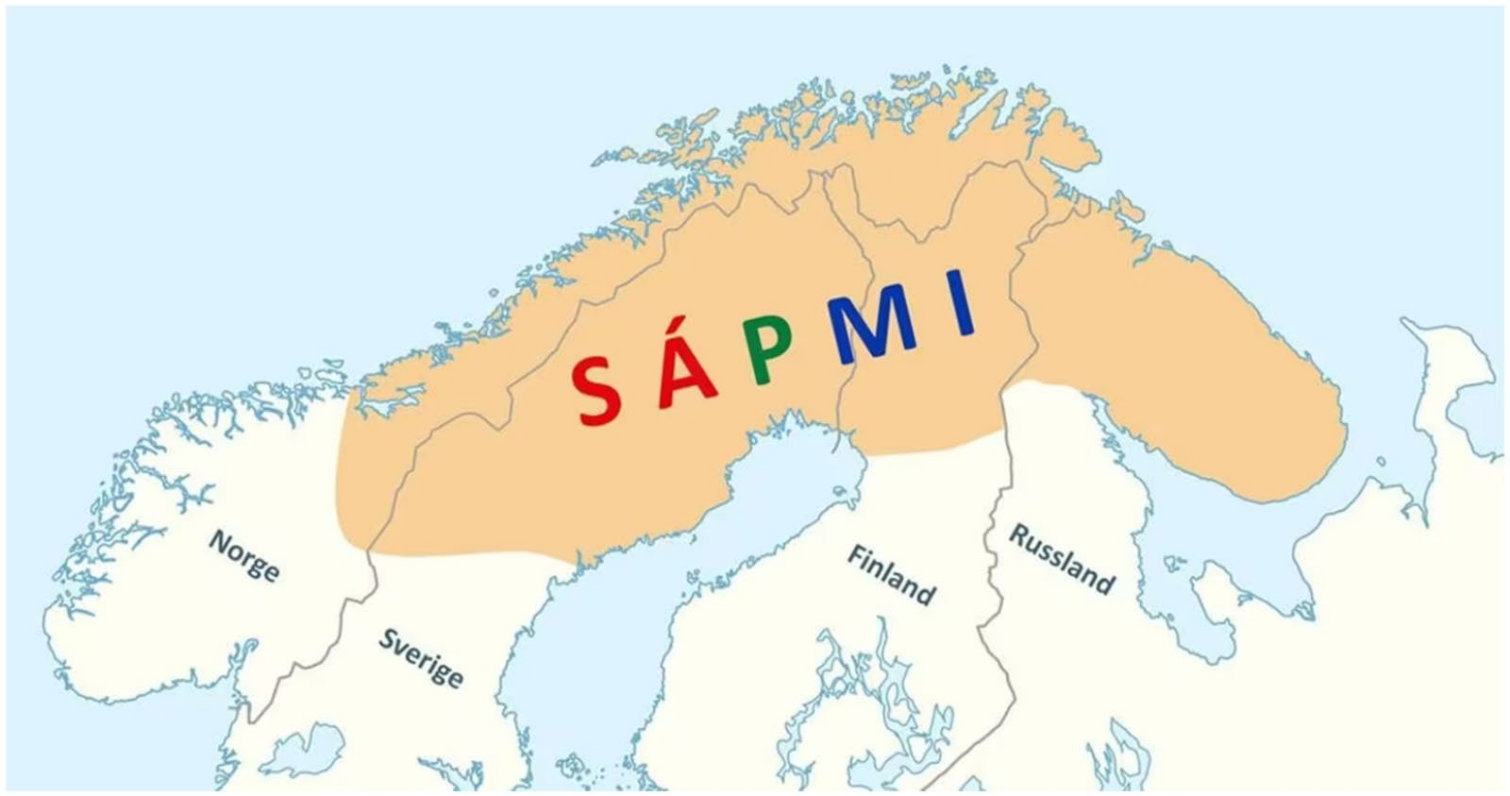
Sápmi. The traditional Sami territory covering Norway, Sweden, Finland and Russia. Map from Finnmark hospital trust CC BY-SA 3.0.

The Sami, the Indigenous people of Sápmi - a region that encompasses the northern parts of Norway, Sweden, and Finland, as well as the Kola Peninsula in Russia (Figure 1) - share a history with other Indigenous populations as being subjected to assimilation, marginalization, and discrimination for generations [6,7]. The systematic assimilation and oppression of the Sami by Norwegian authorities was prominent from 1850 to 1960 and is commonly referred to as The Norwegianization Process. The Norwegianization process did extensive damage to Sami language and culture, and its impact were, and continue to be, grave and evident [7]. Indigenous research in low, middle and also in high-income countries, including Norway, Greenland, USA, Canada, New Zealand and Australia, has shown that being subjected to oppression and forced assimilation is associated with health and socioeconomic disparities, and higher gender- and age-specific morbidity and mortality rates [8–11]. However, the differences in socioeconomic and health related factors between the Sami and the reference population in Norway are found to be smaller compared to other countries with an Indigenous population [1,12]. Still, the Sami population have higher occurrences of symptoms of Angina Pectoris, some cardiovascular risk factors [13,14] including Diabetes 2 and unfavourable weight/height ratios [15]. Differences have also been observed with Sami displaying a higher prevalence of mental distress [9,16], reporting lower levels of self-rated health [17,18], and higher frequencies of experiences of violence and abuse [19,20].

Prolonged traveling distances, lack of available services, and economic disparities are known systemic barriers for use of health care services, as found in a review of literature concerning Indigenous health care access in rural America [21]. In a systematic review focusing on Indigenous peoples residing in Canadian urban areas, demanding interactions due to ethnic discrimination and communication challenges was highlighted as barriers [22]. Barriers for use of health care might also rise from religious belief systems and traditional perspectives on illness and healing [23]. These barriers might rise from personal experiences and from shared stories. Norwegian public health care services are universal, mainly tax founded, and are designed to align with governmental ambitions to ensure accessible high-quality health care across the country, hereby levelling social and economic disparities. Use of insurance based private health care service is currently 13-15% [24]. All residents and registered immigrants in Norway have the right to an assigned General Practitioner (GP) for primary medical care [25]. Referrals to specialized care is administered by the assigned GP. Emergency medical care during office and outside office hours is provided by GPs in decentralized emergency rooms as an integral part of the primary health care service. A comparative study of Sami majority vs. Sami minority areas in northern parts of Norway found no difference in hospitalisation rates in the period 2002–2006 [26]. Further, minor or no significant differences in cancer treatment radiotheraphy have been discovered between Sami majority and minority areas (1999–2008) [27]. A comparison of Sami and non-Sami youth in 2003–2005 found no overall ethnic differences in self-reported use of either school health service, general practitioner nor psychologist/psychiatrist [28]. A study based on data from the SAMINOR 2 Questionnaire Survey (2012), shows no difference between Sami and non-Sami participants in self-reported use of specialised somatic or psychiatric care in adulthood if exposed to childhood emotional abuse [20]. Concerns about a lack of scientific knowledge on Sami health and living conditions was highlighted in a Norwegian official investigation in 1995 (NOU 1995:6). The SAMINOR Study managed by the Centre for Sami Health Research at UiT The Arctic University of Norway (UiT) was launched in 2003, to amend some of the identified knowledge gaps. Although substantial contributions have been made, there are still significant knowledge gaps on Sami health and use of health services, especially the use of primary health care services [29,30]. The aim of this study is to investigate ethnic differences in the use of assigned general practitioner and emergency health care (2012–2019) adjusting for age, level of education and county of residence.

## Methods

**Figure 2.**
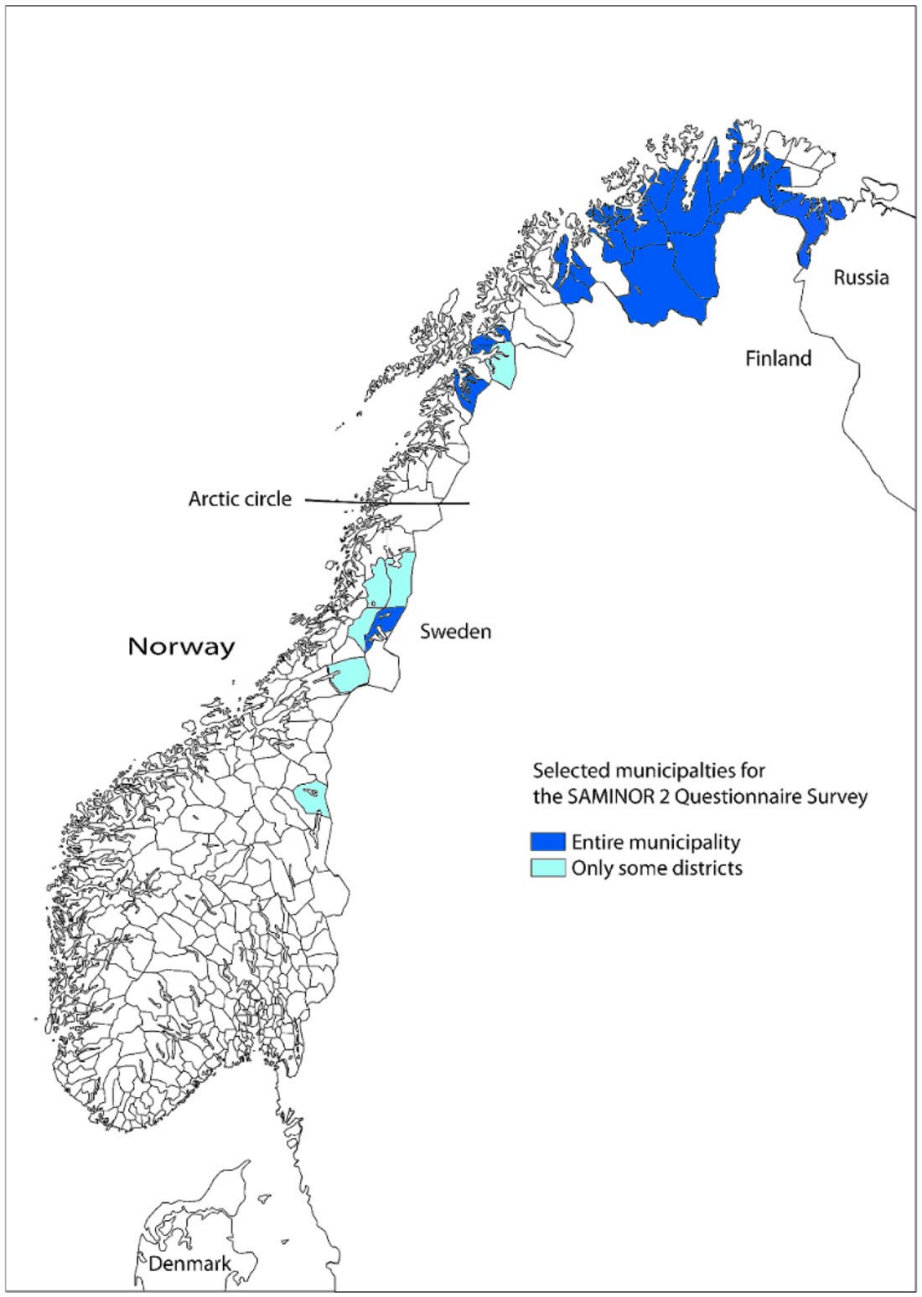
Map of study area. Designed by M. Melhus. Centre for Sami Health Research. UiT the Arctic University of Norway. Shared with permission.

**Figure 3.**
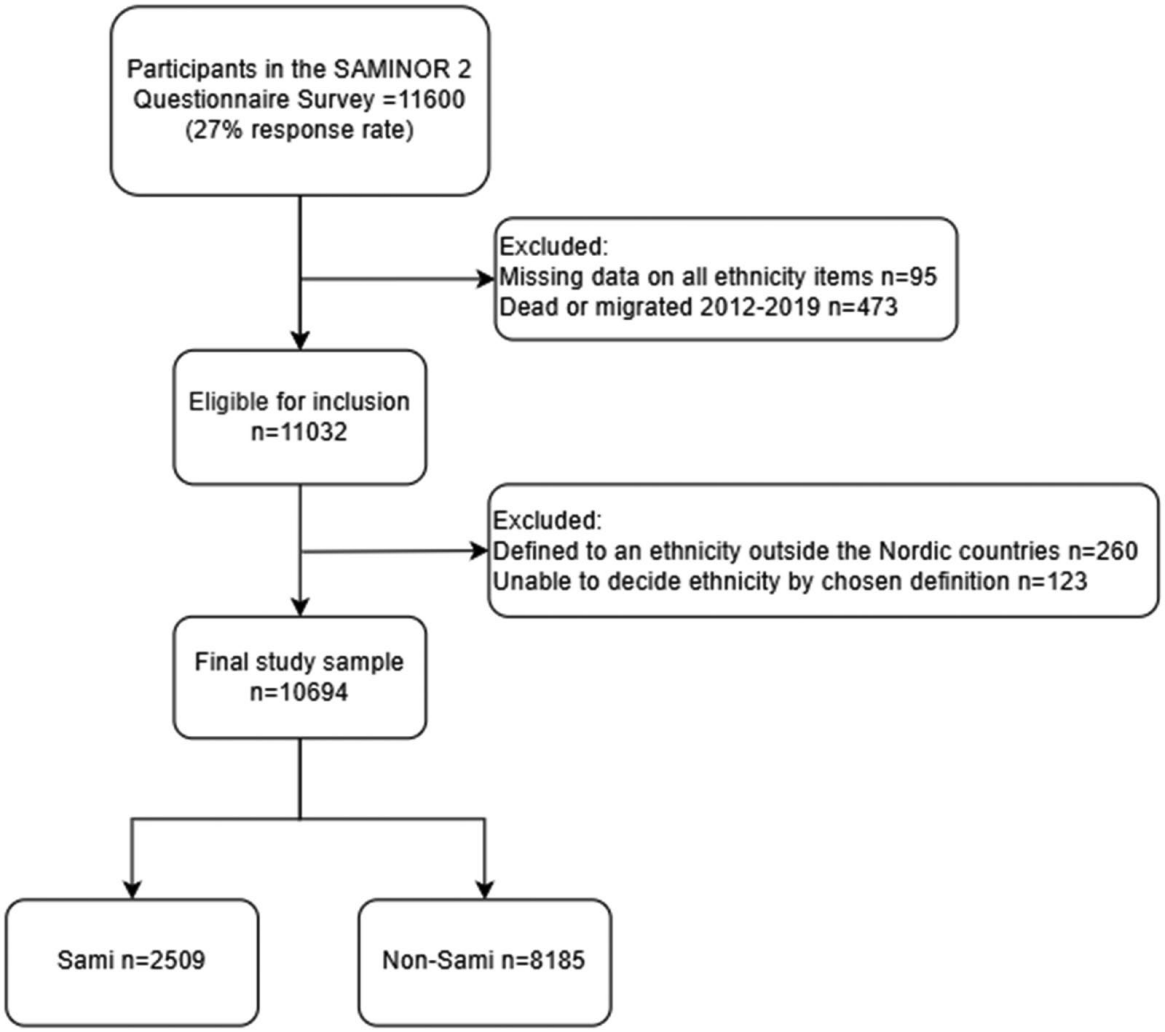
Derivation of the study sample.

The current study sample was derived from the SAMINOR 2 Questionnaire Survey (hereafter referred to as SAMINOR 2), a part of the second wave of the Population-based Study on Health and Living Conditions in Regions with Sami and Norwegian Populations – the SAMINOR Study (2012) (Figure 2). Inhabitants from 25 municipalities aged 18–69 were invited. Details on SAMINOR 2 are described elsewhere [31]. SAMINOR 2 data (study population of 11,600 (27% response rate)) was linked to information on health care utilization from the Norwegian Control and Payment of Health Reimbursements Database (KUHR) and Statistics Norway (SSB) for the study period (2012–2019). The unique Norwegian personal identification number assigned to every citizen and registered immigrant enabled linkage of KUHR and SSB to SAMINOR 2. To protect the participants identity, SSB replaced the personal identification number with an unrelated project specific identifier before data was made available to the authors. The project specific identifier was then used to link data from the three sources. In the current study we excluded participants who emigrated from Norway or died during the study period (2012–2019) (Figure 3). The participants were defined as Sami or non-Sami based on their answers to 9 different questionnaire items on self-defined ethnicity, ethnic background and language affiliation. Responders who could not be defined into any ethnicity because of missing data, incoherent reporting or not to an ethnicity within the Nordic countries (Norway, Sweden, Finland, Denmark, Iceland, Greenland and the Faroe Islands) were excluded. In total, 906 participants were excluded, resulting in an analytical sample of 10 694.

### Outcome variables from the Norwegian Control and payment of health reimbursements database (KUHR)

**Figure 4.**
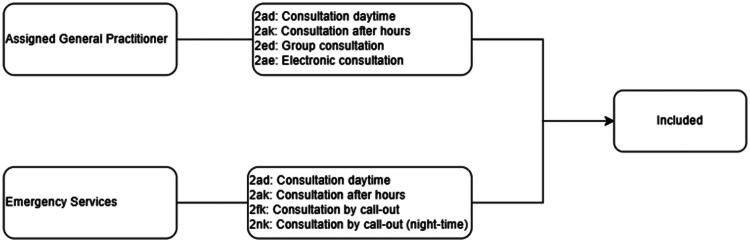
Inclusion of claims from KUHR.

The KUHR Database registers all public health care contacts through reimbursement claims sent by health care providers, indexed using the patient’s personal identification number. KUHR provided the outcome variables by granting access to records on all claims from assigned GPs and GPs affiliated to emergency care for services rendered to study-participants from 2012 to 2019. Each claim included one or more codes in accordance with Norwegian regulations issued by the Ministry of Health and Care Services. All claims coded according to Statistics Norway’s (2024) definition of a GP medical consultation were included. All other claims were disregarded. Each claim was regarded as a single event, even if they contained multiple reimbursement codes, and grouped as GP or Emergency Service consultations as specified in the KUHR database (Figure 4). This way, the outcome variables ‘Use of assigned GP’ contain claims for a) Daytime and Out of Hours consultations, b) Group consultations and c) Electronic consultations (defined as a medical evaluation and response when there is an already established diagnosis). The variables ‘Use of Emergency Care’ contain claims for a) Daytime and Out of Hours consultations, and b) emergency call out response by day or night-time. Both outcomes were calculated separately for each year in the study period (2012–2019).

### Main exposure variable from SAMINOR 2

The main exposure variable in this study was Sami ethnicity. Sami ethnicity was defined based on questions on self-perceived ethnicity, ethnic background and language affiliation. All questions could be answered Norwegian, Sami, Kven (a national ethnic minority group not defined as Indigenous) or Other, and multiple answers were possible. Other ethnicity, ethnic background, and language affiliation could be described in a free text field. Participants were defined as Sami if the answer was ‘Sami’ to at least one of the questions ‘What do you consider yourself to be’, or ‘What ethnic background do you have’? in addition to confirming at least one Sami family language affiliation (‘What language(s) do/did you, your parents or your grandparents speak at home?’). All others were defined as non-Sami.

### Additional SAMINOR 2 variables

Length of education was used as a proxy for socioeconomic status and included in the final models to adjust for socioeconomic status. SAMINOR 2 participants were asked to give the total sum of all years of school and/or studies. We categorized the data into two groups corresponding to the Norwegian education system: ≤12 years and >12 years, indicating lower or higher education. If no total sum was provided, the participant was excluded from the statistical models (*n* = 113). Information on gender (dichotomous variable) and age (continuous variable) and municipality of residence (nominal variable) in 2012 was also obtained from SAMINOR 2. Gender was used to stratify the analysis to explore the impact of ethnicity within the same sex. Age was included in the model as a covariate as increasing age may influence health and healthcare utilization.

We grouped the municipalities by their respective county (as per 2012) as follows: Finnmark county: (Karasjok, Kautokeino, Porsanger, Tana, Nesseby, Lebesby, Alta, Loppa and Kvalsund, Sør-Varanger). Troms county: (Kåfjord, Kvænagen, Storfjord, Lyngen, Skånland and Lavangen). Nordland/Trøndelag: Due to the small number of participants in the three counties Nordland (Tysfjord, Evenes, Hattfjelldal, Grane, Narvik), Nord-Trøndelag (Røyrvik, Namskogan, Snåsa) and Sør-Trøndelag (Røros), these counties were grouped together. Alta and Sør-Varanger have populations of >10,000. All other municipalities have populations ranging from approximately 450 (Røyrvik) to 4000 (Porsanger). We explored use of healthcare stratified by county before including County of residence in the final model as a co-variate.

### Statistics Norway variables

The Norwegian Population Register, governed by the Norwegian Tax Administration, contains information of everyone who resides or have resided in Norway. Data from the register is also available from Statistics Norway (SSB). SSB provided year of emigration and/or immigration, and year of death. SSB also provided the key to link all data sources. Linkage was done using unique Norwegian personal identification numbers replaced with an unrelated project specific identifier before data was made available to the authors.

### Statistical analyses

The analysis was pre-planned prior to the processing of the data, based on the study protocol. All analyses were stratified by gender. Descriptive characteristics are reported as frequency (percent) or mean (standard deviation (SD)). Pearson’s Chi-square test was used to compare distributions of categorical sample characteristics. Significant differences in mean age and number of consultations between the groups was assessed using the two-sample t-test, taking into account Levene’s test for equality of variances. The analyses were done in a repeated cross-sectional design, analysing the number of consultations within each year from 2012 to 2019 separately. We examined the relationship between the outcomes and Ethnicity, Age, Length of education, and County of residence. The general linear model *Poisson loglinear* was used for exploring the rate of GP-consultations and *Negative binominal with log link* was used for the rate of emergency health care consultations. The Negative binomial model was used to account for overdispersion, which was found when analysing use of emergency services by calculating the ratio of variance to the mean. To ensure consistency, we applied the same models and adjusted for the same variables known to influence health care utilization in both models. Results from the loglinear models are reported as Incidence Rate Ratios (IRR) with 95% confidence intervals (CI). Level of significance was set at *p* = 0,05 for all analyses. Hence, if the 95% confidence interval included 1, the result was not considered statistically significant. All IRRs and 95% CIs are rounded to two decimals. This means that CIs like 0.999–1.015 and 1.003–1.015 both may appear to include 1 even if the latter does not. We will consider cases with lower or upper confidence limit of =1.00 as non-significant in order not to overinterpret the results.

Interactions between county of residence and Sami ethnicity were tested by including two interaction terms: Sami × Nordland/Trøndelag and Sami × Finnmark, with Troms county serving as the reference category. Because of observed interaction for some of the years analysed, we considered stratifying on county to explore within-county ethnic differences in the number of health care consultations. Stratification did however lead to some strata lacking statistical strength because of few included participants, and the effect sizes of the interaction terms were small and not consistent from year to year. Therefore, we reverted to our original strategy of adjusting for county of residence considering it as a mediating factor. To investigate whether living in the urban municipalities Alta or Sør-Varanger influenced the conclusions for Finnmark county, sensitivity-analyses were conducted for these two municipalities combined, referred to as ‘Urban Finnmark’. Data from Narvik was collected solely in a rural area within the municipality and is hence not defined as urban. Results from the loglinear models are reported as Incidence Rate Ratios (IRR) with 95% confidence intervals (CI). Level of significance was set at *p* = 0.05 for all analyses. All statistical analyses were done using IBM SPSS Statistics (Version 29.0).

## Ethics

The SAMINOR 2 Questionnaire Survey is approved by the Norwegian Data Protection Authority. All participants gave informed consent to participate when returning the questionnaire. The current study’s Data Protection Impact Assessment was conducted by the Norwegian Agency for Shared Services in Education and Research (Sikt) (741006/23), in collaborations with the project’s PI and approved by the Data Protection Officer at UiT The Artic University of Norway. The Regional Committee for Medical and Health Research Ethics in Northern Norway (REK-Nord) determined that ethical approval was not required (604937). The Ethical Committee for Sami Health Research granted a collective Sami consent to the current project (23/3073-3).

## Results

The final study population included 10,694 individuals: 5986 women (28.9% Sami) and 4708 men (22.7% Sami). Sample characteristics are shown in Table 1. Women (mean age 46.7) were significantly younger than men (mean age 49.5) (*p* < 0.001). Sami women (mean age 45.9) were significantly younger than non-Sami women (mean age 47.0) (*p* < 0.01), but no such difference was found between Sami men (mean age 49.6) and non-Sami men (mean age 49.5) (*p* = 0.40). A higher percentage of Sami women (65.8) than non-Sami women (59.7) reported having more than 12 years of education (*p* < 0.001). There was no ethnic difference in the length of education in men (Table 1).

**Table 1.** Sample characteristics.

	*N* = 10,694	Women *n* = 5986 (56.0%)			Men *n* = 4708 (44.0%)		
		Sami *n* = 1439 (24.0)	Non-Sami *N* = 4547 (76.0)		Sami *n* = 1070 (22.7)	Non-Sami *n* = 3638 (77.3)	
	Mean (SD)	Mean (SD)	Mean (SD)	*p* ^a^	Mean (SD)	Mean (SD)	*p* ^a^
*Age*	47.9 (13.64)	45.9 (13.92)	47.0 (13.71)	<0.01	49.6 (13.23)	49.5 (13.35)	0.40
	***n* (%)**	***n* (%)**	***n* (%)**	*p* ^b^	***n* (%)**	***n* (%)**	*p* ^b^
*County*				<0,01			
*Nordland and Trøndelag*	1206 (11.2)	82 (5.7)	557 (12.2)		66 (6.2)	501 (13.8)	
*Troms*	2179 (12.4)	164 (11.4)	1004 (22.1)		167 (15.6)	841 (23.1)	
*Finnmark*	7308 (68.4)	1192 (82.9)	2985 (65.7)		837 (78.2)	2294 (63.1)	
*Urban Finnmark* ^c^	4535 (27.9)	320 (26.9)	2272 (76.1)	<0.01	208 (24.9)	1735 (75.6)	<0.01
*Rural Finnmark* ^d^	2773 (40.5)	872 (73.1)	713 (23.9)		629 (75.5)	559 (24.4)	
*Education ^e^*				<0.01			0.06
*≤ 12 years*	4464 (42.2)	485 (34.2)	1815 (40.3)		567 (53.4)	1802 (50.1)	
*>12 years*	6117 (57.8)	933 (65.8)	2685 (59.7)		494 (46.6)	1796 (49.9)	

Abbreviations: p: p-value; SD: Standard deviation. a: independent samples Student’s T-test. b: Chi-square test. c: Urban Finnmark consists of two municipalities: the Alta municipality (largest city in Finnmark) with >21.000 inhabitants and the Sør-Varanger municipality (including the city of Kirkenes) with >10.000 inhabitants. d: Rural Finnmark consists of the remaining municipalities in Finnmark included in the SAMINOR 2 Questionnaire Survey. e: 107 individuals had missing information on length of education.

### GP consultations

**Figure 5.**
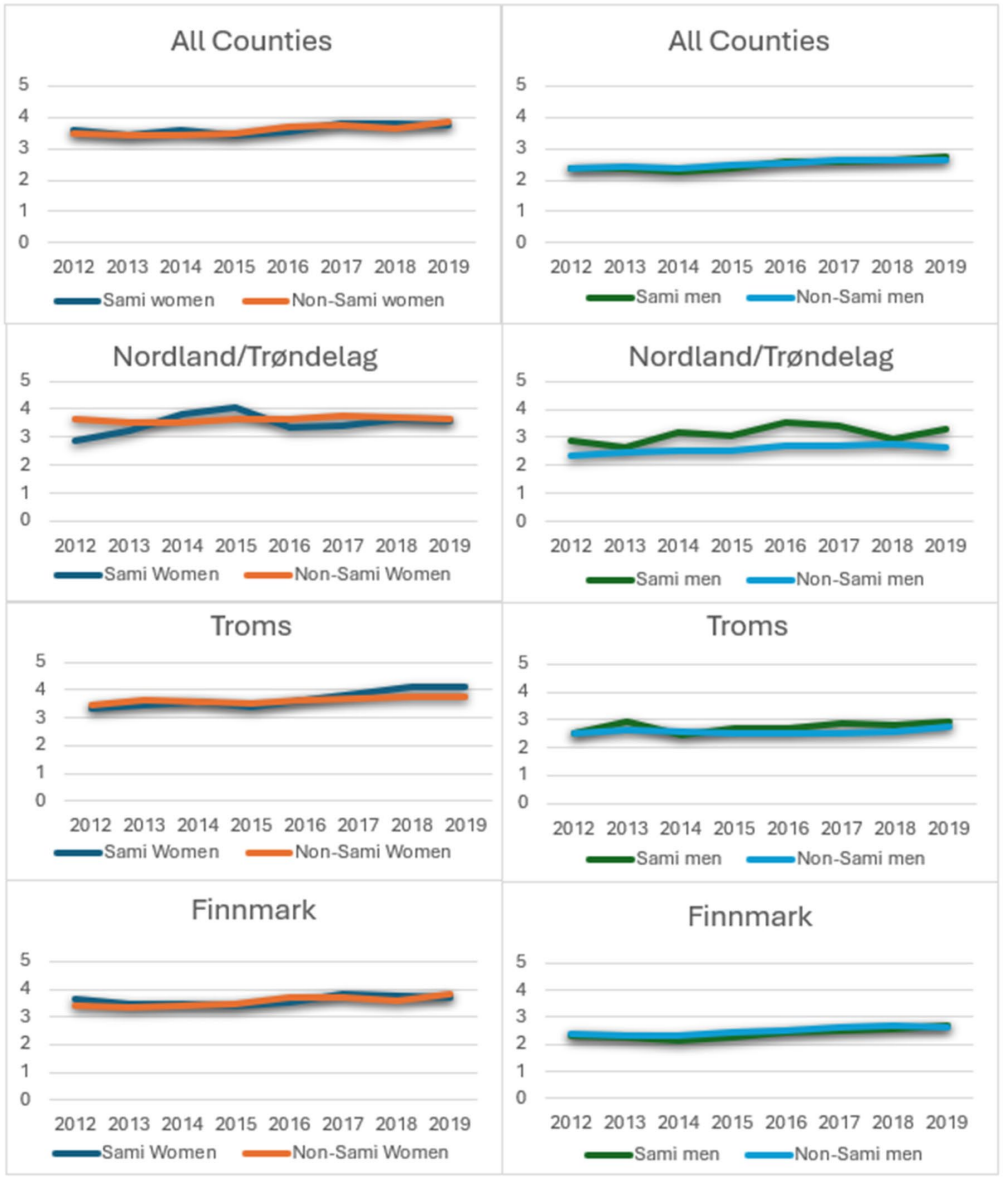
Mean number of General Practitioner consultations in Sami and Non-Sami men and women. Total and by county.

A significant difference in the mean number of GP consultations was observed between women and men across the study period (2012–2019), with women consistently having higher means. Stratified by gender, there were no significant differences in the frequency of GP consultations between Sami and non-Sami neither for women nor men (data not shown). When analyzed by county, significant ethnic differences in mean number of GP consultations emerged in Nordland/Trøndelag only, and only for some of the years analyzed (Figure 5): Sami women had fewer consultations than non-Sami women in 2012 (Sami: 2.86, non-Sami: 3.66, *p* < 0.01 (data not shown). Sami men had more consultations than non-Sami men in 2016 (Sami: 3.53, non-Sami: 2.71, *p* = 0.03. and in 2019 (Sami: 3.29, non-Sami: 2.62, *p* = 0.04, data not shown).

The initial Poisson regression model, with ethnicity as the sole variable (crude analysis), showed that Sami ethnicity was generally not a significant variable for predicting differences in GP consultations (Table 2): In 2016, treating non-Sami women as the reference group (IRR = 1), Sami women had a significantly lower incidence (IRR: 1.05, 95% CI: 1.02–1.08). In men (non-Sami men as the reference group IRR = 1), the crude analysis indicated Sami ethnicity increased the incidence rate for GP-consultations to 1.05 in 2014 (95% CI: 1.00–1.09), while the result in 2015 was border-significant (IRR= 1.04 (95% CI: 1.00–1.09)) with a p-value of 0.06 (data not shown).

**Table 2.** Mean number of GP and emergency care consultations in women and men 2012–2019 (all counties).

	General Practitioner consultations			Emergency Care consultations		
Women	Sami	Non-Sami		Sami	Non-Sami	
	Mean (SD)	Mean (SD)	p^a^	Mean (SD)	Mean (SD)	p^a^
2012	3.58 (3.46)	3.47 (3.43)	0.16	0.52 (1.35)	0.31 (0.85)	<0.01
2013	3.45 (3.67)	3.41 (3.67)	0.44	0.54 (1.37)	0.30 (0.78)	<0.01
2014	2.59 (3.12)	3.45 (3.59)	0.28	0.54 (1.42)	0.31 (0.85)	<0.01
2015	3.46 (3.57)	3.50 (3.68)	0.37	0.58 (1.09)	0.32 (0.83)	<0.01
2016	3.56 (3.80)	3.70 (3.86)	0.09	0.57 (1.27)	0.32 (0.38)	<0.01
2017	3.80 (3.84)	3.73 (3.98)	0.29	0.45 (1.14)	0.29 (0.90)	<0.01
2018	3.79 (3.94)	3.67 (3.84)	0.16	0.42 (1.12)	0.30 (0.94)	<0.01
2019	3.76 (3.86)	3.86 (3.76)	0.46	0.40 (1.04)	0.30 (0.90)	<0.01
	General Practitioner consultations			Emergency Care consultations		
Men	Sami	Non-Sami		Sami	Non-Sami	
	Mean (SD)	Mean (SD)	*p* ^a^	Mean (SD)	Mean (SD)	*p* ^a^
2012	2.38 (3.28)	2.40 (3.11)	0.41	0.42 (0.99)	0.25 (0.66)	<0.01
2013	2.36 (3.44)	2.41 (3.29)	0.31	0.41 (1.03)	0.25 (0.68)	<0.01
2014	2.28 (3.23)	2.40 (3.17)	0.15	0.37 (0.87)	0.23 (0.62)	<0.01
2015	2.36 (3.05)	2.47 (3.23)	0.15	0.35 (0.77)	0.27 (0.78)	<0.01
2016	2.59 (3.30)	2.53 (3.29)	0.30	0.38 (0.84)	0.26 (0.74)	<0.01
2017	2.61 (3.28)	2.64 (3.40)	0.39	0.30 (0.66)	0.25 (0.70)	0.03
2018	2.64 (3.18)	2.65 (3.35)	0.50	0.30 (0.69)	0.23 (0.69)	<0.01
2019	2.77 (3.27)	2.66 (3.22)	0.17	0.33 (0.75)	0.24 (0.62)	<0.01

Abbreviations: p: p-value; SD: Standard deviation. a: p-value calculated by independent samples Student’s t-test.

**Table 3.** Crude and adjusted incidence rate ratios for the use of General Practitioner and Emergency health care of Sami vs non-Sami, stratified by gender, 2012–2019.

			General Practitioner		Emergency Health Care	
			Model^a^		Model^b^	
*Year*	Gender	Ethnicity	Crude IRR (95% CI)	Adj IRR (95% CI)^c^	Crude IRR (95% CI)	Adj IRR (95% CI)^c^
*2012*	Women	Sami	1.03 (1.00–1.06)	0.98 (0.94–1.03)	1.67 (1.50–1.86)	1.80 (1.61–2.01)
		Non-Sami	1	1	1	1
	Men	Sami	1.00 (0.95–1.04)	0.98 (0.94–1.03)	1.72 (1.51–1.96)	1.79 (1.56–2.05)
		Non-Sami	1	1	1	1
*2013*	Women	Sami	1.01 (0.97–1.04)	1.02 (0.99–1.06)	1.79 (1.61–1.99)	1.83 (1.64–2.04)
		Non-Sami	1	1	1	1
	Men	Sami	0.98 (0.98–1.04)	1.08 (0.98–1.19)	1.67 (1.46–1.90))	1.66 (1.45–1.90)
		Non-Sami	1	1	1	1
*2014*	Women	Sami	1.01 (0.98–1.04)	1.03 (0.99–1.06)	1.75 (1.57–1.95)	1.85 (1.66–2.07)
		Non-Sami	1	1	1	1
	Men	Sami	0.96 (0.91–1.00)	0.97 (0.88–1.08)	1.58 (1.37–1.81)	1.60 (1.39–1.84)
		Non-Sami	1	1	1	1
*2015*	Women	Sami	0.98 (0.95–1.02)	1.00 (0.97–1.03)	1.50 (1.35–1.68)	1.53 (1.37–1.71)
		Non-Sami	1	1	1	1
	Men	Sami	0.96 (0.92–1.00)	1.06 (0.96–1.17)	1.30 (1.13–1.49)	1.27 (1.11–1.46)
		Non-Sami	1	1	1	1
*2016*	Women	Sami	0.95 (0.93–0.99)	0.96 (0.93–0.99)	1.79 (1.61–2.00)	1.82 (1.63–2.03)
		Non-Sami	1	1	1	1
	Men	Sami	1.03 (0.99–1.07)	1.03 (0.98–1.07)	1.47 (1.29–1.69)	1.48 (1.29–1.70)
		Non-Sami	1	1	1	1
*2017*	Women	Sami	1.01 (0.98–1.04)	1.02 (0.99–1.05)	1.58 (1.41–1.76)	1.65 (1.47–1.85)
		Non-Sami	1	1	1	1
	Men	Sami	0.99 (0.95–1.03)	1.15 (1.04–1.26)	1.21 (1.04–1.39)	1.20 (1.04–1.39)
		Non-Sami	1	1	1	1
*2018*	Women	Sami	1.03 (1.00–1.06)	1.04 (1.01–1.08)	1.42 (1.27–1.59)	1.51 (1.35–1.70)
		Non-Sami	1	1	1	1
	Men	Sami	1.00 (0.96–1.04)	1.08 (0.98–1.19)	1.31 (1.13–1.51)	1.43 (1.24–1.65)
		Non-Sami	1	1	1	1
*2019*	Women	Sami	0.99 (0.96–1.02)	0.99 (0.96–1.02)	1.34 (1.20–1.51)	1.41 (1.26–1.59)
		Non-Sami	1	1	1	1
	Men	Sami	1.04 (1.00–1.08)	1.03 (0.99–1.07)	1.34 (1.20–1.51)	1.41 (1.25–1.89)
		Non-Sami	1	1	1	1

Abbreviations: IRR: Incidence rate ratio; CI: Confidence interval. Adj: adjusted. a: Poisson log linear model. b: Negative binominal model with log-link. c: adjusted for age (continuous variable), length of education (≤12 years/>12years) and county of residence (Nordland/Trøndelag, Troms, Finnmark) at SAMINOR 2-participation.

Adding the covariates age, education, and county of residence to the model altered some conclusions. In women, Sami ethnicity was a significant predictor in the crude result for 2012 but not in the adjusted model (adjIRR: 0.98, 95% CI: 0.94–1.03). The non-significant crude result for Sami ethnicity in men in 2017 was significant in the adjusted model (adjIRR: 1.15, 95% CI: 1.04–1.26), whereas the significant crude result in 2019 became non-significant (adjIRR: 1.03, 95% CI: 0.99–1.07). Overall, the inclusion of age, education, and county of residence in the model did not consistently alter the conclusions regarding ethnicity and GP consultations.

### Emergency health care

**Figure 6.**
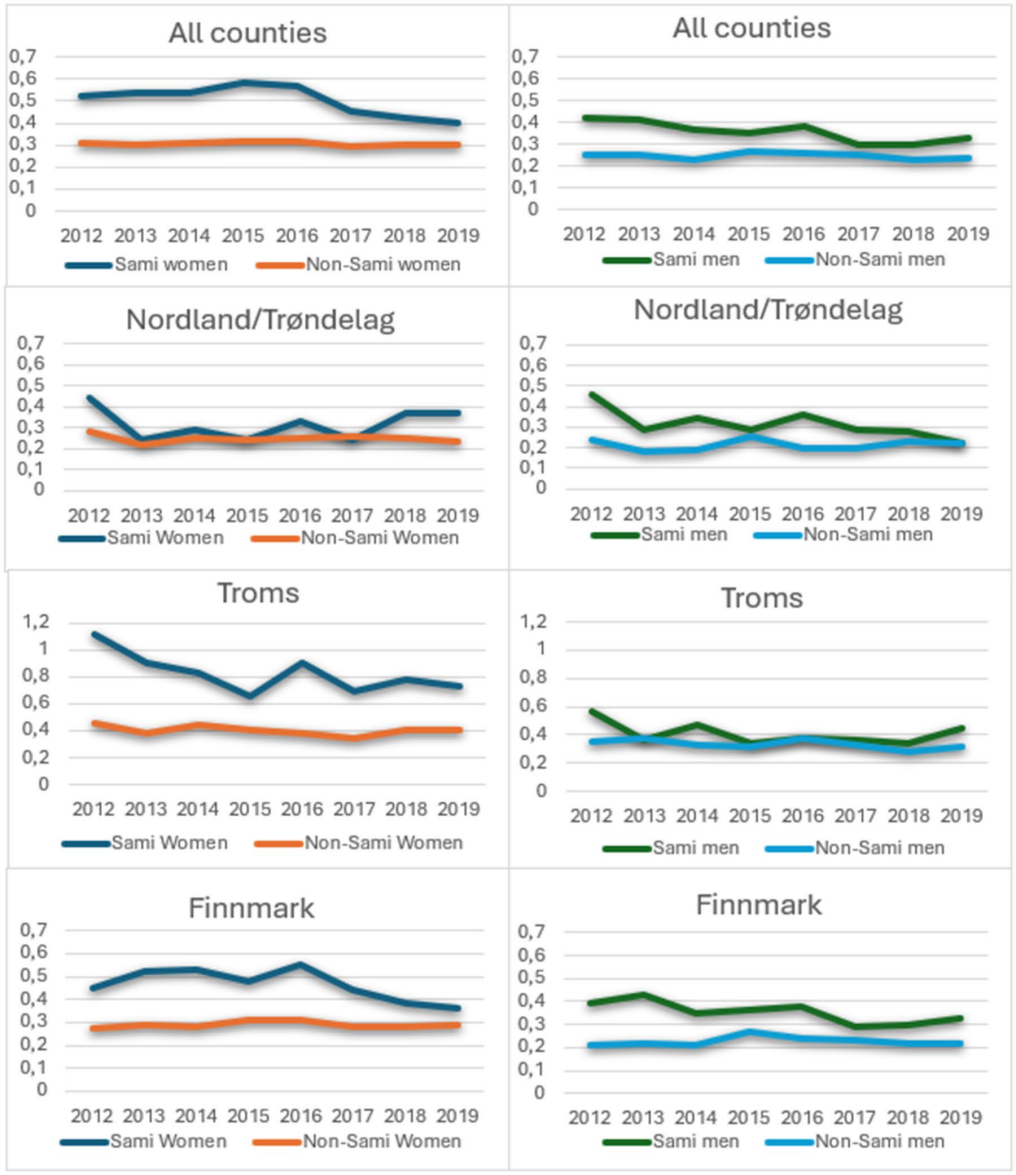
Mean number of Emergency Care consultations in Sami and Non-Sami men and women. Total and by county.

The mean number of emergency consultations differed significantly between women and men, as well as between Sami and non-Sami individuals. Sami women consistently had the highest mean across the study period compared to non-Sami women and men in general (Figure 6). The differences in the frequency of emergency health care consultations between Sami and non-Sami women and between Sami and non-Sami men were statistically significant throughout the study period (all p-values <0.01 (data not shown)). County-level analyses revealed no significant ethnic differences in the mean number of emergency care consultations among women in Nordland/Trøndelag. However, in Troms, Sami women had more than twice the number of emergency health care consultations compared to non-Sami women in 2012 (Sami: 1.12, non-Sami: 0.45) and in 2013 (Sami: 0.90, non-Sami: 0.38). The differences in Troms remained significant except for 2014 (*p* = 0.06 data not shown). Sami women in Finnmark consistently had significantly more emergency health care consultations than non-Sami women. Among men, Sami individuals had an allover higher mean number of emergency consultations than non-Sami (Figure 6). However, stratified by county, these results were statistically significant only for 2012 in Nordland/Trøndelag (*p* = 0.03 data not shown), in Troms for the years 2012, 2014, and 2019 with *p* = 0.04 *p* = 0.03 and *p* = 0.03, respectively (data not shown). In Finnmark county Sami men had significantly higher mean number of emergency consultations than non-Sami men for the entire study period.

Due to observed overdispersion, for emergency care a negative binomial regression model was used to adjust ethnicity for the co-variates age, years of education, and county of residence. In the crude model, Sami ethnicity was associated with a significantly increased incidence for emergency consultations across all years in the study period for both genders, ranging from 34% to 79% in women and 21% to 72% in men (Table 2). Adjustments consistently enhanced the IRR for Sami ethnicity in women. In men the IRR dropped in 2013 from 1.67 to 1.66, in 2015 from 1.30 to 1.27, and from 1.21 to 1.20 in 2017 without changing the conclusion of a significant positive association between Sami ethnicity and use of emergency care.

### Analyses by county of residence

**Figure 7.**
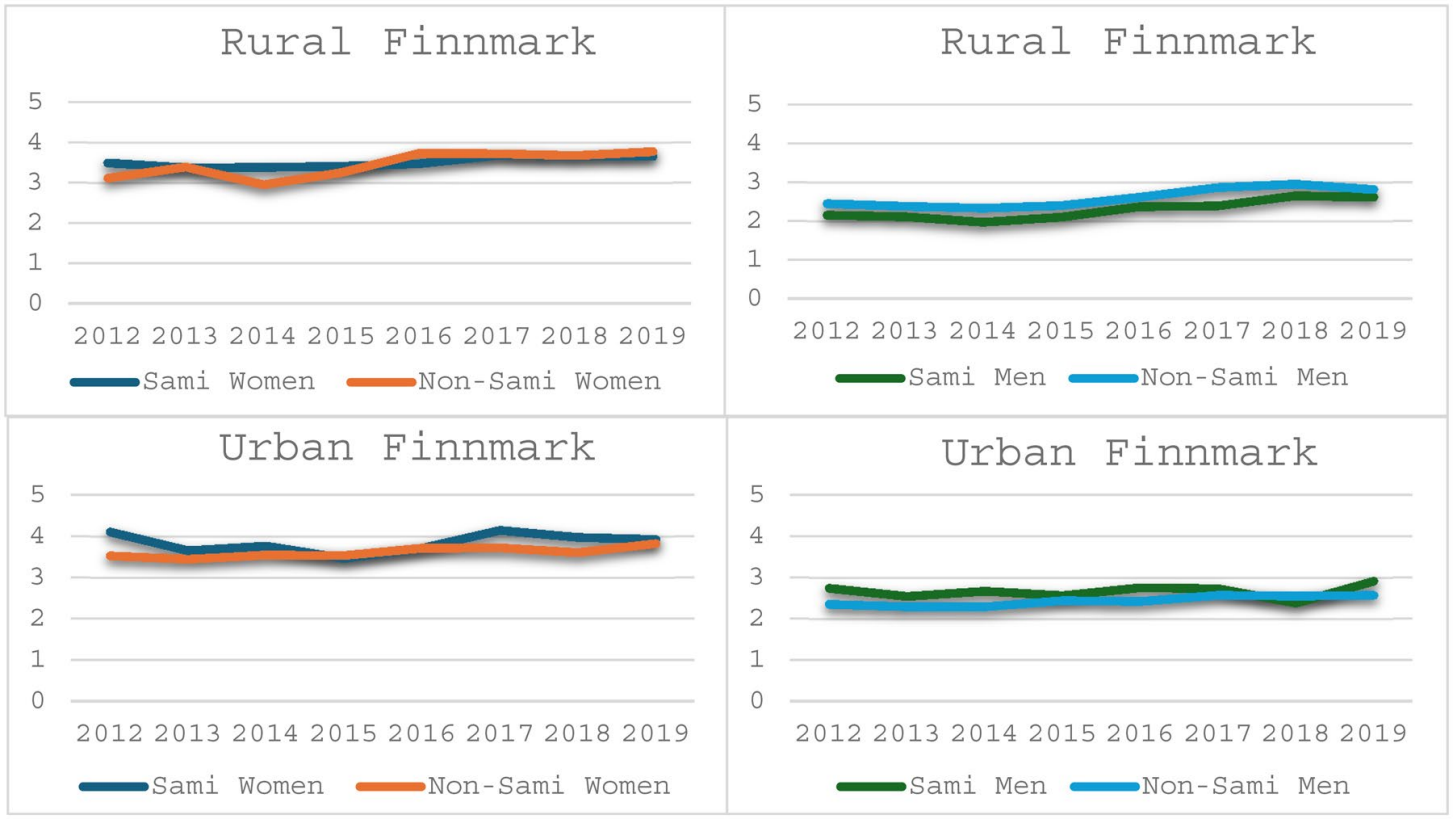
Mean number of Assigned GP consultations in Sami and Non-Sami men and women in rural and urban municipalities within Finnmark county.

**Figure 8.**
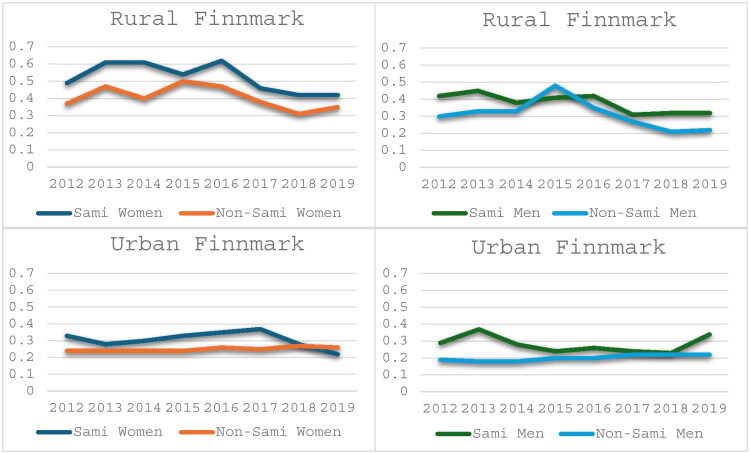
Mean number of Emergency Health Care consultations in Sami and Non-Sami men and women in rural and urban municipalities within Finnmark county.

More than 80% of all Sami and 64.9% of all non-Sami participants lived in Finnmark county (Table 1). Within Finnmark, 60.6% of Sami women and 41.2% of Sami men lived outside of Alta and Sør-Varanger (Urban Finnmark). The corresponding numbers for non-Sami were 15.5% for women and 15.4% for men. Focusing on the area Urban Finnmark (Figures 7 and 8), the mean number of GP consultations were significantly higher among Sami than non-Sami women in 2012 (*p* < 0.01) and 2017 (*p* = 0.04), and Sami men had significantly more GP consultation in 2012 (*p* < 0.01) and 2014 (*p* = 0.04) (Figure 7). Emergency health care consultations were significantly higher among Sami women in 2012 (*p* = 0.02), 2016 (*p* = 0.04), and 2017 (*p* = 0.01) and among Sami men in 2012–2014 and 2019, with p-values ranging from 0.02–0.03

### Sensitivity analysis

Excluding Urban Finnmark from the Poisson or Negative binominal regression analyses did not alter the results for Sami ethnicity in women or men in the crude or adjusted model for Finnmark county (data not shown).

## Discussion

The aim of this study was to explore whether there are ethnic (Sami versus non-Sami) differences in the use of GP and emergency health care. The study provides important information about the use of health care in areas with multiethnic populations by linking SAMINOR 2 to official registers and databases. Contrary to prior assumptions, difference in use of assigned GP was not found. However, a consistent significant disparity was observed in use of emergency health care, with Sami women and men having higher mean number of consultations than non-Sami women and men. The perception that Sami individuals only seek healthcare when strictly necessary has persisted as a widely accepted truth, both in public discourse and among governing bodies [26,29]. Our study does not provide evidence to support these perceptions or the findings of lower utilization of primary healthcare services among Sami than non-Sami presented by Fuggeli in 1991. Instead, the higher use of emergency care observed among Sami individuals is to our knowledge a novel finding in a Norwegian setting.

### Geographical and social determinants of health barriers

Our findings align with international studies showing higher emergency healthcare utilization among Indigenous populations compared to majority populations. Globally, inequalities in use of health care are often linked to geographical as well as social determinants of health (SES) barriers [32]. Indigenous communities frequently reside in remote areas with limited access to primary healthcare, leading to delayed treatment and increased reliance on emergency services. However, the Sami and non-Sami populations in this study reside in the same rural areas, sharing similar access to healthcare and education. This shared environment minimizes geographical and SES disparities, making direct comparisons to international studies challenging. In Norway, where both GP and emergency healthcare services are organized at the municipal level and often co-located, geographical barriers are further reduced. Additionally, Norway’s universal and tax-funded healthcare system is designed to minimize socioeconomic disparities. Therefore, geographical and SES barriers are unlikely to explain the higher emergency healthcare utilization observed among Sami individuals in this study.

### A higher burden of disease

The main reasons for the use of emergency care in Norway are primarily acute conditions, injuries, or severe worsening of illnesses that require immediate medical care [33]. A greater burden of chronic illnesses could act as a contributing factor explaining the increased emergency healthcare utilization among Sami individuals. Globally, Indigenous populations often experience higher rates of chronic conditions such as diabetes, cardiovascular disease, respiratory illnesses, chronic pain, and mental health problems [1,2], which may lead to more frequent health crises. Among the Sami, previous SAMINOR studies have reported higher prevalence of non-communicable diseases [13–15] mental health distress, and experiences of violence and abuse [16,20]. A systematic review assessing health care access and use found that First-Nations peoples had higher hospital admission rates for injuries due to falls, injuries associated to assaults, motor vehicle accidents and poisoning than non-First nations peoples [2]. Studies in Norway and Sweden have pointed to Sami, and Sami men in particular being involved in the primary industries and hence being more subjected to accidents and acute illness [34,35]. While these physical, mental, and occupational health disparities likely contribute to the increased use of emergency healthcare services, the overall health differences between Sami and non-Sami in Norway are relatively small and cannot fully account for the observed disparities.

### Historical trauma, cultural norms and communication barriers

A higher utilization of emergency healthcare among Sami may be influenced by several other interconnected factors, including cultural and systemic factors. Studies have shown that Indigenous populations often face barriers related to trust in healthcare providers [36]. For instance, a Swedish cross-sectional study including reindeer-herding Sami and a non-Sami control group found that Sami participants were significantly more likely to report a lack of confidence in primary healthcare providers [34]. Similarly, a systematic review identified distrust as a factor negatively impacting healthcare utilization among Indigenous men in New Zealand, Australia, Canada, and the United States [37]. A lack of culturally appropriate care and mistrust of the healthcare system, rooted in historical injustices, racism, and assimilation policies, may discourage Sami individuals from seeking timely medical attention.

In addition to mistrust, the use of traditional healing may impact on health care utilization. Traditional healing along with a lack of trust in conventional medicine and health care providers is found in studies included in a scoping review of use of traditional Indigenous medicine in North America [23]. The use of traditional medicine providers was far more common among Sami than non-Sami participants in the SAMINOR 1 Survey in 2003–2004 [38]. Also, data from the Tromsø Study (2015–2016) showed higher use of traditional medicine providers among Sami than non-Sami [39], aligning with a Swedish study findings showing use of conventional medicine to be prevalent among some Sami individuals [40].

The use of traditional healing practices may reflect cultural values, such as independence and resilience, that also influence care-seeking behaviours. A study looking at differences in child-rearing between Sami and non-Sami parents found that Sami children are raised to be independent, hardy and execute internal self-control [41]. Internalized factors influencing care-seeking behaviours are also highlighted in the research literature included in a scoping review on Aboriginal traditional parenting [42]. Cultural norms emphasizing resilience and self-reliance, encapsulated in the concept of ‘weathering the storm’ [41,43], may lead to under-communication of symptoms, causing GPs to underestimate the severity of a condition.

The Sami population reports encountering cultural and language barriers that hinder effective communication and reduce the motivation to contact a GP for health concerns [36]. Such significant barriers have been identified in a narrative review of care access barriers among Canada’s Indigenous populations, particularly pointing to a lack of qualified healthcare personnel with language and cultural competence [44]. Sami expressions of illness, which are often less explicit and more imagery-based, may hinder effective communication with healthcare providers, leading to delays in diagnosis and treatment [29,45]. There is a notable scarcity of Sami-speaking GPs and healthcare personnel with adequate knowledge of Sami culture, even in core Sami regions [46]. This shortage contributes to language barriers and broader communication challenges, which are further exacerbated by limited understanding of Sami traditions, cultural views on illness and healing, and a lack of trust in the healthcare system.

## Strengths and limitations

A major strength of this study is the utilization of the national KUHR database, which encompasses all reimbursement claims from primary healthcare providers. This ensures that the database provides a reliable and accurate reflection of the actual number of consultations in general practice and emergency services. Since Sami ethnicity is not recorded in any national registers, comparisons between Sami and non-Sami populations are only feasible when this information is obtained from external sources, such as the SAMINOR Study. By combining these two datasets, the study enables the exploration of factors that would otherwise remain inaccessible. Additionally, the large sample size, drawn from multiple municipalities, offers a robust representation of Sami settlement areas in Northern Norway. Other variables reflecting health status and socioeconomic besides those explored in this study could act as confounders or effect mediators and should be further investigated. With a response rate of 27% in SAMINOR 2, the results should however be interpreted with caution. A low response rate might increase the risk of introducing selection bias in the sample, which means the sample may no longer be representative. This in turn can lead to skewed results not applicable to the target population.

Ethnicity can be categorized by other definitions than the one used in this study. Alternative definitions could have altered the number of participants in the Sami and non-Sami groups, enlarging or declining the number of participants defined as Sami. Independently of the definition used when dividing people into groups based on characteristics given in a questionnaire, information bias by misclassification may occur. In this project we have adhered to the classification of ethnicity in other SAMINOR 2 studies [9,14,16].

## Conclusion

In contrast to international and national research on healthcare services utilization among Indigenous peoples, our study found no differences in the use of general practitioners between Sami and non-Sami populations. However, we did find a significantly higher average use of emergency medical services, suggesting a greater need for acute medical treatment among the Sami compared to non-Sami. The higher utilization of emergency healthcare services among the Sami population is likely influenced by a combination of factors, including a greater burden of illness, mistrust of the healthcare system, cultural norms and communication challenges. Further research should investigate the underlying and likely complex factors driving the observed differences.

## Data Availability

Data cannot be shared publicly due to the nature of the materials and adherence to the ethical and legal contract with the SAMINOR Project Board, UiT the Arctic University of Norway, Statistics Norway (SSB) and the Control and Payout of Health Reimbursement Database (KUHR).
